# Brown adipocyte mineralocorticoid receptor deficiency impairs metabolic regulation in diet-induced obese mice

**DOI:** 10.1016/j.jlr.2023.100449

**Published:** 2023-09-20

**Authors:** Chu-Mao Chen, Xiao-Qian Meng, Hong Zhu, Ting Liu, Yuan Liu, Lu-Jun Zhou, Guo-Dong Zhu, Xiao-Bei Chen, Xu-Guang Guo, Sheng-Zhong Duan

**Affiliations:** 1 Department of Clinical Laboratory Medicine, Guangdong Provincial Key Laboratory of Major Obstetric Diseases, Guangdong Provincial Clinical Research Center for Obstetrics and Gynecology, The Third Affiliated Hospital of Guangzhou Medical University, Guangzhou, China; 2 Laboratory of Oral Microbiota and Systemic Diseases, Shanghai Ninth People’s Hospital, College of Stomatology, Shanghai Jiao Tong University School of Medicine, Shanghai, China; 3 National Center for Stomatology, National Clinical Research Center for Oral Diseases, Shanghai Key Laboratory of Stomatology, Shanghai, China; 4 Hongqiao International Institute of Medicine, Shanghai Tongren Hospital/Faculty of Basic Medicine, Key Laboratory of Cell Differentiation and Apoptosis of Chinese Ministry of Education, Shanghai Jiao Tong University School of Medicine, Shanghai, China; 5 Department of Oncology, Guangzhou Geriatric Hospital, Guangzhou, China; 6 Department of Infectious Diseases, Renmin Hospital of Wuhan University, Wuhan, China; 7 Department of Clinical Medicine, The Third Clinical School of Guangzhou Medical University, Guangzhou, China; 8 Guangzhou Key Laboratory for Clinical Rapid Diagnosis and Early Warning of Infectious Diseases, King Med School of Laboratory Medicine, Guangzhou Medical University, Guangzhou, China; 9 Department of Teaching Management, The Third Affiliated Hospital of Guangzhou Medical University, Guangzhou, China; 10 Shanghai Key Laboratory of Hypertension, Shanghai Institute of Hypertension, Shanghai, China

**Keywords:** adipose tissue, brown, mineralocorticoid receptor, obesity, glucose metabolism, insulin resistance, energy metabolism, inflammation

## Abstract

Activation of brown adipose tissue (BAT) contributes to energy dissipation and metabolic health. Although mineralocorticoid receptor (MR) antagonists have been demonstrated to improve metabolism under obesity, the underlying mechanisms remain incompletely understood. We aimed to evaluate the role of BAT MR in metabolic regulation. After 8 weeks of high-fat diet (HFD) feeding, BAT MR KO (BMRKO) mice manifested significantly increased bodyweight, fat mass, serum fasting glucose, and impaired glucose homeostasis compared with littermate control (LC) mice, although insulin resistance and fasting serum insulin were not significantly changed. Metabolic cage experiments showed no change in O_2_ consumption, CO_2_ production, or energy expenditure in obese BMRKO mice. RNA sequencing analysis revealed downregulation of genes related to fatty acid metabolism in BAT of BMRKO-HFD mice compared with LC-HFD mice. Moreover, H&E and immunohistochemical staining demonstrated that BMRKO exacerbated HFD-induced macrophage infiltration and proinflammatory genes in epididymal white adipose tissue (eWAT). BMRKO-HFD mice also manifested significantly increased liver weights and hepatic lipid accumulation, an increasing trend of genes related to lipogenesis and lipid uptake, and significantly decreased genes related to lipolytic and fatty acid oxidation in the liver. Finally, the level of insulin-induced AKT phosphorylation was substantially blunted in eWAT but not liver or skeletal muscle of BMRKO-HFD mice compared with LC-HFD mice. These data suggest that BAT MR is required to maintain metabolic homeostasis, likely through its regulation of fatty acid metabolism in BAT and impacts on eWAT and liver.

Obesity increases the risk of various diseases, including type 2 diabetes, nonalcoholic fatty liver disease, and cardiovascular diseases, posing serious threats to human health (1, 2, 3). An important mechanism underlying the threat of obesity is to impair glucose homeostasis and insulin sensitivity (4). Many strategies have been developed to control obesity and its comorbidities, but limited success has been achieved because of the complex regulation of obesity (5, 6).

Brown adipose tissue (BAT) plays an important role in regulating physiology mainly in rodents and human infants (7, 8). It dissipates chemical energy and releases heat by fatty acid oxidation to protect against hypothermia and obesity (9). BAT transplantation limits weight gain, increases energy expenditure (EE), and improves insulin sensitivity in genetic or diet-induced obese mice (10, 11). Conversely, removal of BAT or deletion of uncoupling protein 1 (UCP1) aggravates obesity and/or glucose intolerance in mice (12). Moreover, the secretory functions of BAT in metabolic regulation have been increasingly appreciated (13). For instance, the lack of BAT-derived angiopoietin-like 4 has been shown to positively regulate lipoprotein lipase activity and control glucose metabolism (14). Another study has demonstrated that glucose and insulin tolerance are profoundly impaired in obese mice that have received transplantation of BAT from interleukin-6 KO mice (15). Therefore, BAT and its secretory factors may provide novel strategies to intervene in obesity and related metabolic dysfunction. Although BAT is crucial for rodents and newborns in metabolic regulation, its function in adult humans is not fully understood and is likely to be less metabolically significant than in mice (16).

Recent studies have highlighted the important role of mineralocorticoid receptor (MR) in controlling obesity and metabolic syndrome (17, 18). MR is a nuclear receptor and a crucial drug target for hypertension and heart failure (19). Notably, MR blockade with eplerenone has shown metabolic benefits and more EE in obese mice (20, 21, 22). Several studies have illustrated that deletion of adipocyte MR exerts minor to modest improvement on obesity-induced metabolic disorders. It is reported that MR deficiency of mature adipocytes does not affect glucose metabolic homeostasis or insulin resistance under a high-fat diet (HFD) and/or high-sucrose diet (23, 24). However, Ferguson *et al.* (25) have shown that adipose tissue-specific MR KO reduces adiposity, hepatic steatosis, and increases EE without influencing insulin sensitivity in mice fed with HFD containing 0.2% cholesterol. Conversely, overexpression of adipocyte MR strongly exacerbates metabolic dysregulation (26). These studies did not differentiate the roles of MR between white adipose tissue (WAT) and BAT. The specific function of BAT MR in metabolic regulation has not been explored.

In the current study, we explored the role of BAT MR in obesity and metabolic homeostasis. First, we generated BMRKO mice and tested the impacts of BAT MR deficiency on HFD-induced obesity, glucose intolerance, and insulin resistance. Then we explored the effect of BMRKO on energy metabolism using metabolic cages. Subsequently, we determined the effect of BMRKO on gene expression in BAT, inflammation in WAT, and hepatic steatosis. Finally, we determined the impacts of BMRKO on insulin sensitivity of major insulin target tissues.

## Materials and methods

### Animals

BAT MRKO and LC mice were established by crossing MR-LoxP mice (27) with UCP1-Cre mice (28) on a C57BL/6 background. All animals were maintained under a specific pathogen-free facility with 12 h light/dark cycles and were free to access food and water. All the animals used in this study were male mice. Mice were fed with a normal chow diet (NCD) or a HFD (D12492, 60 kcal% fat; Research Diet, Inc) from the age of 6 to 14 weeks. Bodyweight (BW) was measured every week during the experiment. All animal protocols were approved by the Institutional Review and Ethics Board of Shanghai Ninth People’s Hospital, Shanghai Jiao Tong University School of Medicine.

### Glucose tolerance test and insulin tolerance test

A glucose tolerance test (GTT) was performed in mice at the age of 14 weeks by intraperitoneal injection of a glucose solution at 1 g/kg BW after 16 h fasting. After 1 week of recovery, an insulin tolerance test (ITT) was conducted in mice fasted for 5 h by insulin injection at 1 U/kg BW. Blood glucose was measured with a glucometer at 0, 15, 30, 60, and 120 min after intraperitoneal injections. The area under the curve of GTT and ITT was calculated in GraphPad Prism software (version 8.0.2; GraphPad Software, Inc).

### Body composition and EE measurement

Body composition was measured using a nuclear magnetic resonance-based body composition spectroscopy (Minispec LF50; Bruker, Germany) (29). Briefly, mice were individually immobilized in a restrainer and placed into the spectroscopy. Then the NMR signals of all protons in mice were acquired and analyzed by Minispec LF50 to obtain data for absolute fat and lean mass.

Before the EE measurement, metabolic cage system was calibrated with N_2_ and CO_2_ according to the manufacturer’s instructions. Mice were housed individually to acclimate for 24 h. Subsequently, the data of whole-body O_2_ consumption (VO_2_), CO_2_ production (VCO_2_), and EE were measured and acquired by multiplexed Promethion systems (Sable Systems, NV). The experiments were carried out for more than 24 h using indirect calorimetry at room temperature. The data were measured at 5 min intervals. In addition, daily food intake was measured during the experiments. The metabolic data were normalized to lean body mass as described previously (30, 31). Mice were maintained under a 12 h light/dark cycle and fed with NCD or HFD. Food and water were available ad libitum.

### Tissue sample collection, fasting serum glucose, insulin, and hepatic lipid measurement

Sixteen-week-old mice were anesthetized and sacrificed after 6 h of fasting. Parts of interscapular BAT, WAT, and liver were fixed in paraformaldehyde or snap-frozen in liquid nitrogen and then stored at −80°C for further analyses. Blood was collected in tubes without anticoagulation, and serum samples were obtained after centrifugation at 3,500 rpm/min for 20 min. The fasting serum insulin level was determined using a Mouse Ultrasensitive Insulin ELISA kit (ALPCO, Salem, NH). Fasting serum glucose level was quantified by a glucose oxidase method according to the manufacturer’s instructions (Applygen, Beijing, China). Total hepatic triglycerides and cholesterol were determined using commercial assay kits (Applygen).

### Histology and crown-like structure analysis

Interscapular BAT, WAT, and liver were fixed in 4% paraformaldehyde overnight at 4°C. Tissues were embedded in paraffin followed by alcohol dehydration, and 5 μm sections were prepared for H&E staining and immunohistochemistry (IHC). The IHC staining was performed as previously described with modifications (32). Briefly, the rehydrated sections were boiled in Tris-EDTA antigen retrieval buffer (G1206; Servicebio, Wuhan, China) at 65°C for 8 min and 55°C for 7 min. Then 3% hydrogen peroxide was used to quench endogenous peroxidases. The tissue sections were incubated with anti-Mac2 (GB11246; Servicebio) at 4°C overnight, subsequently HRP-labeled secondary antibodies (GB23303; Servicebio), and then 3,3′-diaminobenzidine staining (K5007; DAKO, CA). Images were captured using a Leica microscope. Crown-like structure (CLS) in epididymal WAT (eWAT) was analyzed as described precisely (33). Three to five cross-sections of one mouse were chosen for analysis using ImageJ software (version 1.52, National Institutes of Health). The total number of CLS per section was counted, and macrophage infiltration of adipose tissue was quantified as CLS per square millimeter.

### Western blotting

Liver, WAT, and skeletal muscle were harvested in HFD-fed mice 10 min after insulin injections. BAT, eWAT, and inguinal WAT (iWAT) of NCD-fed mice were used to determine the protein level of MR. Total proteins were extracted using RIPA buffer containing protease and/or phosphatase inhibitor cocktail. The proteins were separated by SDS-PAGE after quantification with a BCA assay. Thereafter, the isolated protein was transferred onto a polyvinylidene fluoride membrane, incubated with primary antibodies against AKT (9271; Cell Signaling Technology), p-AKT^(Ser473)^ (9272; Cell Signaling Technology), p-GSK3β^(Ser9)^ (9336; Cell Signaling Technology), GSK3β^(Ser9)^ (9315; Cell Signaling Technology), and MR (41912; Abcam) and then secondary antibodies. Finally, the signals were detected using HRP substrates (34580; ThermoFisher Scientific). The protein levels were quantified by using ImageJ and normalized to total protein or α-tubulin.

### Quantitative RT-PCR

Total RNA was extracted from BAT, eWAT, iWAT, liver, adrenal gland, kidney, and hypothalamus using Trizol reagent. The concentration and quality of RNA were determined using NanoDrop Spectrophotometers. Then complementary DNA was synthesized with a commercial kit (Takara, Shiga, Japan). Quantitative PCR was performed with SYBR Green PCR reagents on a LightCycler® 480 system (Roche, Switzerland). Primer sequences are listed in supplemental Table S1. The level of mRNA was normalized to GAPDH expression.

### RNA sequencing analysis

RNA was extracted from BAT of LC-HFD and BMRKO-HFD groups (*n* = 4 per group). A sequencing library was constructed according to the manufacturer's protocol and sequenced on a NovaSeg^TM^ platform (PE150; Illumina, Inc). RNA sequencing data were filtered and mapped to the reference genome using spliced mapping algorithm and then annotated in StringTie software (Department of Computer Science, Johns Hopkins University, Baltimore, MD). Next, the DEsq2 R package was used to identify differentially expressed genes (DEGs) with *P*.adj <0.1. Heatmap was generated to show the hierarchical clustering of DEGs, and a volcano plot was drawn by ggplot R package. The Gene Ontology database was performed in R software for functional and signaling pathway analysis.

### Statistical analysis

All data are presented as mean ± SD. Unpaired Student’s *t*-test was applied to determine the difference between the two groups. Two-way ANOVA was used for comparisons between curves with GraphPad Prism software. *P* < 0.05 was considered statistically significant.

## Results

### Efficient deletion of MR in BAT of BMRKO mice

**Fig. 1:**
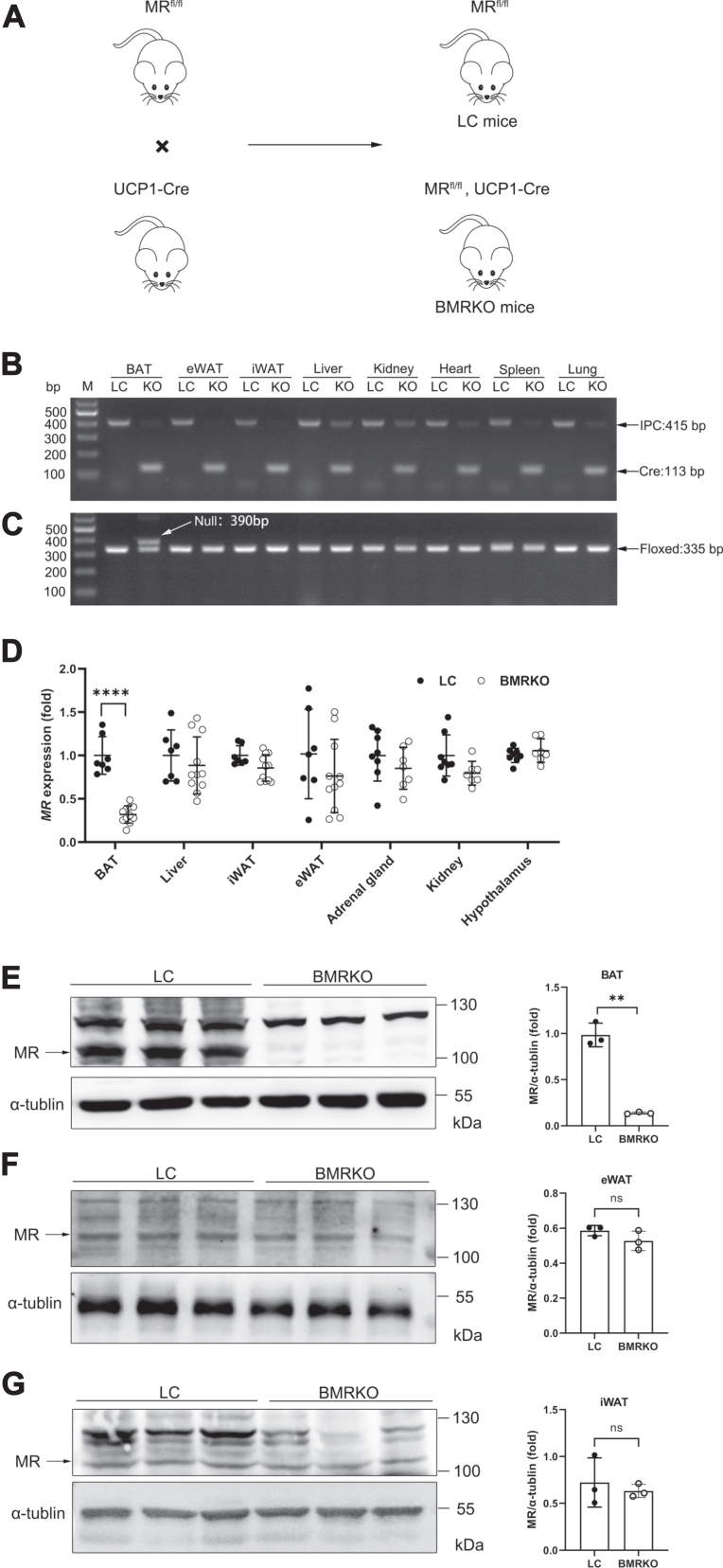
Generation of BMRKO mice. A: The breeding strategy to generate BMRKO mice and LC mice. B: Agarose gel electrophoresis of the PCR product of UCP1-Cre recombinase gene (113 bp) and internal positive control (IPC) in different tissues. C: Agarose gel electrophoresis of the PCR product of MR floxed (335 bp) and null (390 bp) alleles in different tissues. D: RT-qPCR analysis of MR expression in BAT, liver, iWAT, eWAT, adrenal gland, kidney, and hypothalamus. *n* = 7:11 for BAT, liver, iWAT, and eWAT; *n* = 8:7 for adrenal gland, kidney, and hypothalamus. E–G: Western blotting analysis and quantifications of MR protein levels in BAT, eWAT, and iWAT of LC and BMRKO mice. *n* = 3:3. Values are expressed as mean ± SD. ∗∗*P* < 0.01 and ∗∗∗∗*P* < 0.0001.

To explore the function of MR in BAT during obesity, we crossed MR-LoxP mice with UCP1-Cre mice to generate BMRKO mice (Fig. 1A). Results of genotyping PCR demonstrated that the gene of UCP1-Cre recombinase (113 bp) existed in different tissues, including BAT, eWAT, iWAT, liver, kidney, heart, spleen, and lung of BMRKO mice (Fig. 1B). The null (390 bp) allele of MR was detected only in BAT, indicating that UCP1-Cre recombinase was specifically expressed in BAT of BMRKO mice (Fig. 1C). RT-quantitative PCR (qPCR) analysis showed that MR gene expression significantly decreased in BAT (68% reduction) of BMRKO mice compared with LC mice but not in other tissues (Fig. 1D). Consistent with these observations, results of Western blotting analysis illustrated that MR protein was substantially decreased in BAT (86% reduction) but not iWAT and eWAT of BMRKO mice (Fig. 1E–G). These results demonstrated efficient although not complete deletion of MR in BAT of BMRKO mice.

### BAT MR deficiency exacerbates diet-induced obesity and glucose intolerance

**Fig. 2:**
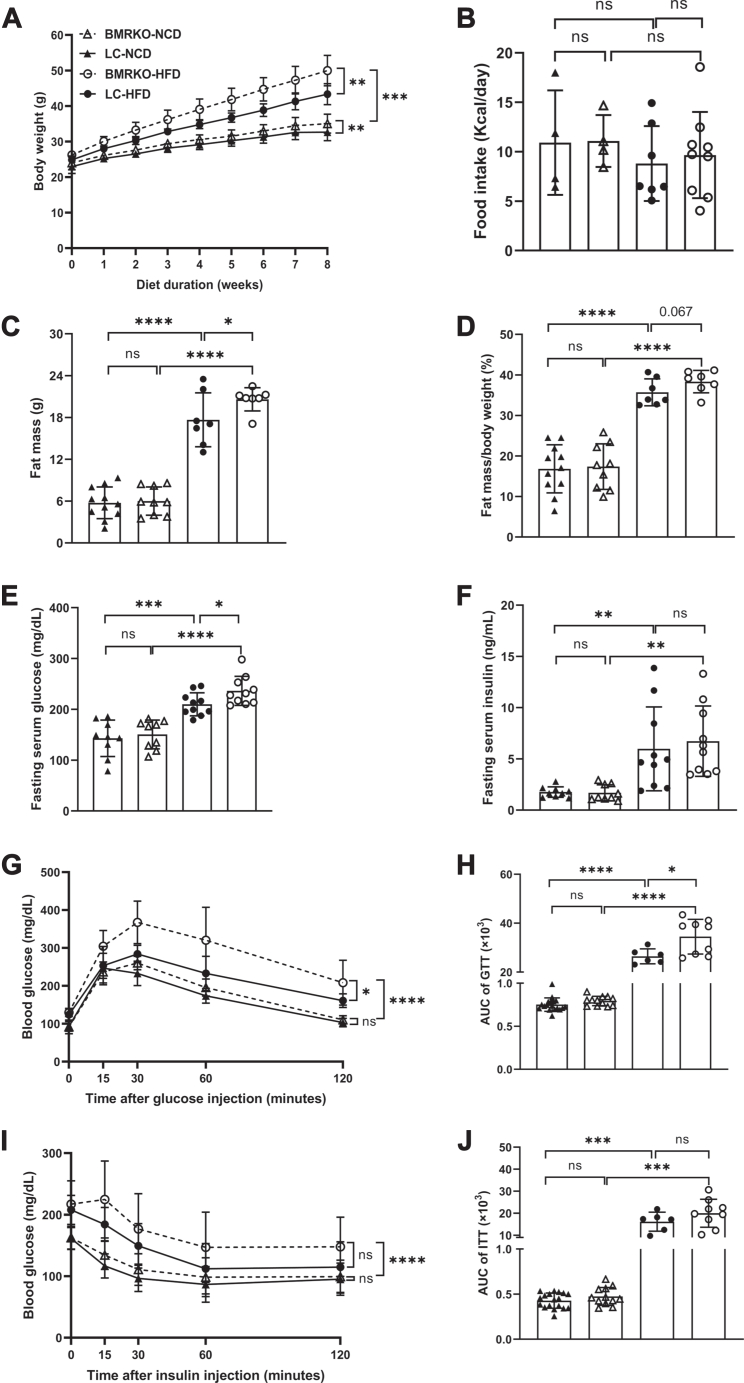
BAT MR deficiency exacerbates diet-induced obesity and glucose intolerance in mice. A: Changes in BW of LC mice and BMRKO mice during 8 weeks of HFD or NCD feeding. *n* = 18:12:6:9. B: Average daily food intake. *n* = 4:4:7:9. C, D: Absolute fat mass and fat mass to BW ratio. *n* = 11:9:7:7. E, F: Fasting serum glucose level and fasting serum insulin level in mice after 8 weeks of HFD and NCD. *n* = 9:9:10:10. G: GTT. *n* = 18:12:6:9. H: The area under the curve (AUC) of GTT. *n* = 18:12:6:9. I: ITT. *n* = 18:12:6:9. J: AUC of ITT. *n* = 18:12:6:9. Values are expressed as mean ± SD. ∗*P* < 0.05, ∗∗*P* < 0.01, ∗∗∗*P* < 0.001, and ∗∗∗∗*P* < 0.0001. ns, not significant.

LC and BMRKO mice were fed with an HFD for 8 weeks to study the impact of BAT MR deficiency on obesity and metabolism. Compared with LC-HFD mice, BMRKO-HFD mice had significantly more BW gain (Fig. 2A). There was also a slight but statistically significant increase in BW of BMRKO-NCD mice compared with LC-NCD mice (Fig. 2A). No significant difference in daily food intake were observed between LC and BMRKO mice under NCD and HFD feeding (Fig. 2B). Significant increase in fat mass and a trend of increase in fat mass/BW ratio (*P* = 0.067) were observed in BMRKO-HFD mice compared with LC-HFD mice (Fig. 2C, D). Fat mass and fat mass/BW ratio were both comparable between BMRKO-NCD mice and LC-NCD mice (Fig. 2C, D). In addition, BMRKO-HFD mice manifested a significant increase in fasting serum glucose but no significant change in insulin levels compared with LC-HFD mice (Fig. 2E, F). Remarkably, GTT showed that BMRKO mice exhibited significantly impaired glucose tolerance compared with LC mice under HFD (Fig. 2G, H). Moreover, ITT demonstrated that BMRKO mice tended to have impaired insulin sensitivity compared with LC mice, particularly under HFD (Fig. 2I, J).

### BAT MR deficiency affects lipid metabolism of BAT in mice fed with HFD

**Fig. 3:**
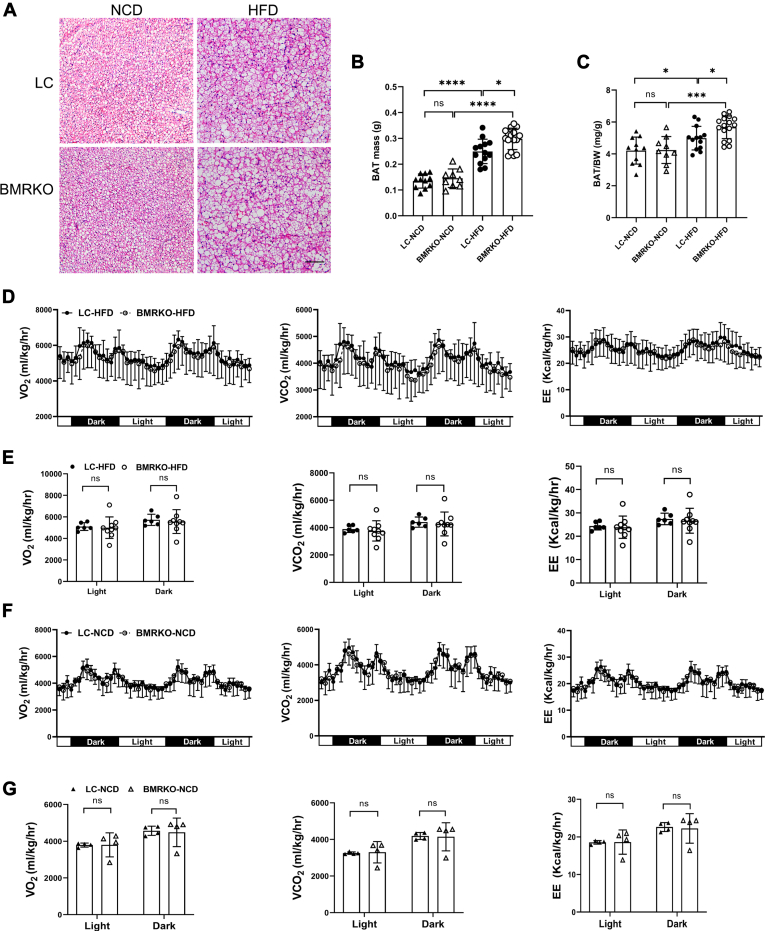
BAT MR deficiency affects BAT lipid metabolism in mice fed with HFD. A: Representative H&E staining of BAT. Scale bar represents 50 μm. B, C: Absolute BAT mass and BAT mass to BW ratio (BAT/BW). *n* = 11:9:13:16. D: Monitoring of VO_2_, VCO_2_, and EE in mice fed with HFD for 8 weeks. Data were normalized to lean mass. E: Quantification of VO_2_, VCO_2_, and EE of mice fed with HFD during light and dark phases. *N* = 6:9. F: monitoring of VO_2_, VCO_2_, and EE in mice fed with NCD for 8 weeks. Data were normalized to lean mass. G: quantification of VO_2_, VCO_2_, and EE of mice fed with NCD during light and dark phases. *n* = 4:4. Values are expressed as mean ± SD. ∗*P* < 0.05, ∗∗*P* < 0.01, ∗∗∗*P* < 0.001, and ∗∗∗∗*P* < 0.0001. ns, not significant.

**Supplemental Figure S1:**
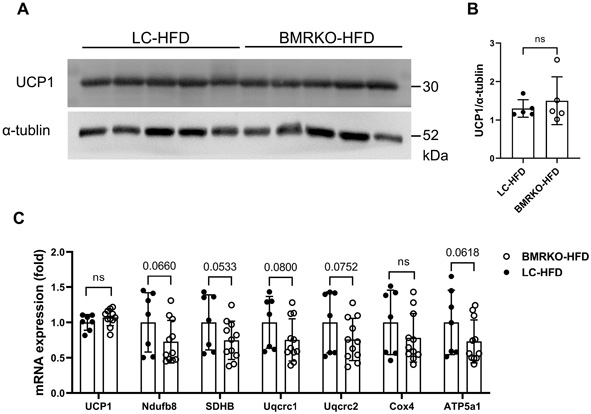
BAT MR deficiency does not affect UCP1 or genes related to BAT respiration under obesity. A: Western blotting analysis of UCP1 in BAT of mice fed with HFD for 8 weeks. B: Quantification of Western blotting results of BAT. *n* = 5:5. C: RT-qPCR analysis of UCP1 and genes related to mitochondrial oxidative phosphorylation in BAT. *n* = 7:11. Values are expressed as mean ± SD. ns, not significant.

We examined the activation of BAT since it is a metabolic organ of dissipating energy (34). H&E staining showed more lipid droplets in BAT of BMRKO-HFD mice compared with LC-HFD mice, suggesting an increase in lipid storage (Fig. 3A). Furthermore, BAT mass and BAT mass to BW ratio were both significantly higher in BMRKO-HFD mice than in LC-HFD mice (Fig. 3B, C). To investigate the impacts of BAT MR deficiency on energy metabolism, whole-body O_2_, VCO_2_, and EE were measured in mice fed with NCD and HFD. No difference was observed in energy metabolism between LC-HFD and BMRKO-HFD mice (Fig. 3D, E). BAT MR deficiency did not affect the expression of UCP1 or genes related to mitochondrial oxidative phosphorylation (*Ndufb8*, *SDHB*, *Uqcrc1*, *Uqcrc2*, *Cox4*, and *ATP5a*) in BAT (supplemental Fig. S1). In addition, there was no difference in VO_2_, VCO_2_, or EE between BMRKO-NCD and LC-NCD mice (Fig. 3F, G).

### BAT MR deficiency affects the expression of genes related to fatty acid metabolism in mice fed with HFD

**Fig. 4:**
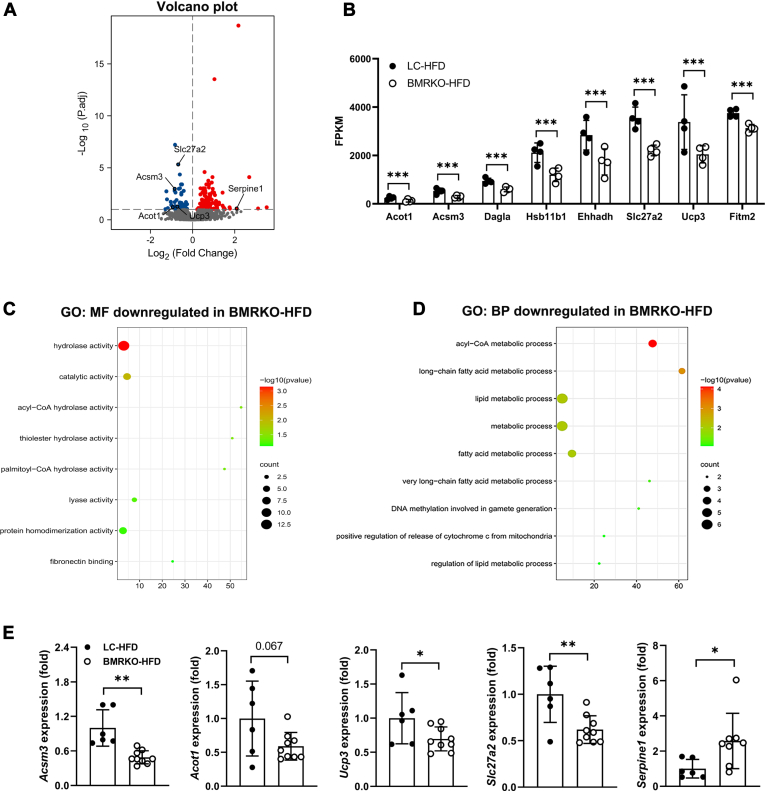
BAT MR deficiency downregulates fatty acid metabolism-related genes in mice fed with HFD. A: Volcano plot of RNA sequencing data showing DEGs in BAT of BMRKO-HFD versus LC-HFD. LC and BMRKO mice were fed with HFD for 8 weeks. Red dots represent upregulated genes, and blue dots represent downregulated genes. B: DEGs related to fatty acid metabolism. *n* = 4:4. C: Gene Ontology (GO) analysis of downregulated genes categorized by molecular function (MF). D: GO analysis of downregulated genes categorized by biological process (BP). E: RT-qPCR validation of DEGs in BAT of mice fed with 8 weeks of HFD. *n* = 6:9. Values are expressed as mean ± SD. ∗*P* < 0.05, ∗∗*P* < 0.01, and ∗∗∗*P* < 0.001. ns, not significant.

To better understand the functions of MR in BAT, we performed RNA sequencing analysis of BAT from mice after 8 weeks of HFD. When compared with BAT of LC-HFD mice, a total of 125 DEGs were upregulated and 62 were downregulated in BMRKO-HFD mice (Fig. 4A). *Serpine 1*, an adipokine in thrombosis and insulin resistance regulation, was substantially upregulated, and genes involved in lipid metabolism, including *Slc27a2*, *Acsm3*, *Acot1*, and *Ucp3*, were dramatically decreased in BAT of BMRKO-HFD mice (Fig. 4A). BAT of BMRKO-HFD mice showed an overall trend of decrease in the expression of genes related to fatty acid metabolism, including *Acot1*, *Acot4*, *Acsm3*, *Hsd11β1*, *Ehhadh*, *Ucp3*, *Slc27a2*, *Dagla*, and *Slc22a5* (Fig. 4B). Moreover, Gene Ontology analysis of the RNA sequencing data showed that the downregulated biological processes and molecular functions in BAT of BMRKO-HFD mice were associated with lipid metabolism and its enzyme activity (Fig. 4C, D). Results of RT-qPCR showed that the expression of *Acsm3*, *Slc27a2*, and *Ucp3* significantly decreased in BAT of BMRKO-HFD mice compared with LC-HFD mice, although the decrease of *Acot1* did not reach statistical significance (Fig. 4G). On the other hand, the expression of *serpine1* significantly increased in BAT of BMRKO-HFD mice (Fig. 4G).

### BAT MR deficiency exacerbates inflammation in epididymal adipose tissues

**Fig. 5:**
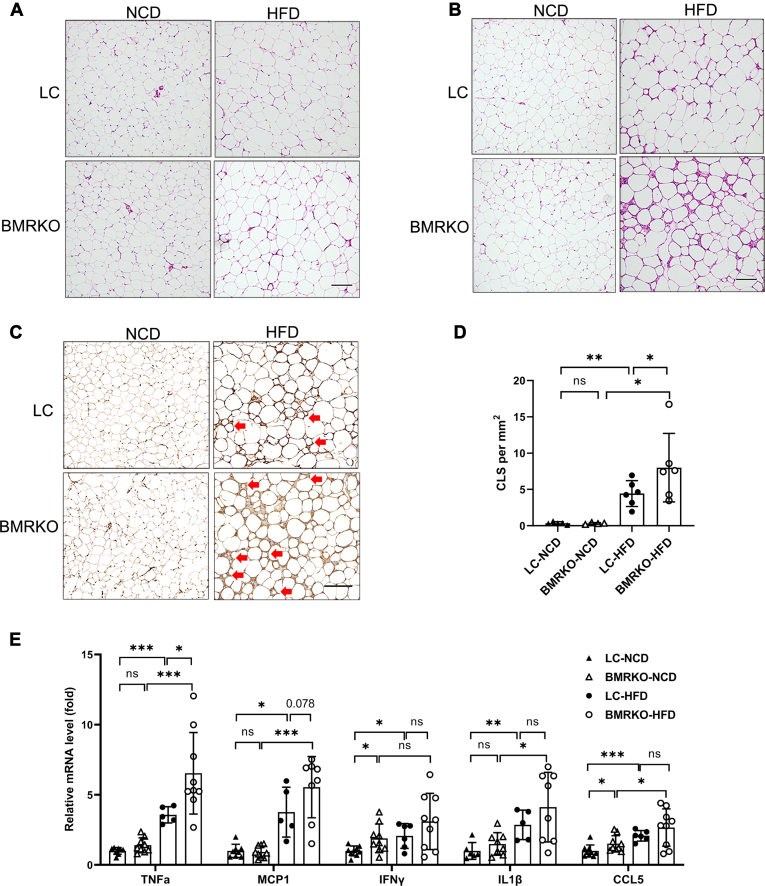
BAT MR deficiency exacerbates inflammation in epididymal adipose tissues. A, B: Representative H&E staining sections of iWAT (A) and eWAT (B) in mice fed NCD and HFD. C: Representative immunohistochemical staining sections of Mac2 in eWAT; CLSs were indicated by red arrows. D: Quantification of CLS in eWAT from mice fed with NCD and HFD. *n* = 5:4:6:6. E: RT-qPCR analysis of inflammatory genes in eWAT. Scale bars represent 100 μm. *n* = 8:9:6:9. Values are expressed as mean ± SD. ∗*P* < 0.05, ∗∗*P* < 0.01, and ∗∗∗*P* < 0.001. ns, not significant.

**Supplemental Figure S2:**
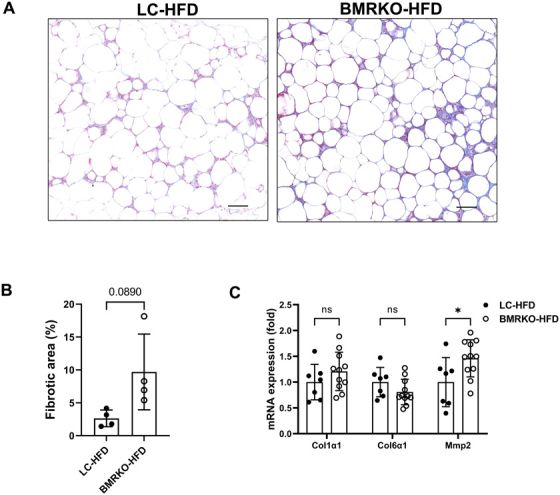
BAT MR deficiency tends to enhance fibrosis in eWAT under HFD. A: Representative images of Masson’s trichrome staining in eWAT of mice fed with HFD. Scale bar: 100 μm. B: Quantification of fibrotic areas in eWAT of obese mice. *n* = 4:4. C: RT-qPCR analysis of fibrotic genes in eWAT. *n* = 7:11. Values are expressed as mean ± SD. ∗*P* < 0.05. ns, not significant.

Systematic insulin resistance and glucose homeostasis are associated with adipose inflammation (35, 36). The H&E staining visibly illustrated more inflammatory cell infiltration in WATs, particularly eWAT, of BMRKO-HFD mice than LC-HFD mice (Fig. 5A, B). IHC staining of macrophages by Mac2 further demonstrated that eWAT of BMRKO-HFD mice had significantly more CLS than that of LC-HFD mice (Fig. 5C, D). Results of RT-qPCR revealed a significant increase of expression of proinflammatory factor *TNFα* and a trend of increase of *MCP1* in eWAT of BMRKO-HFD mice compared with LC-HFD mice (Fig. 5E). However, the expression of other proinflammatory genes, including *IFNγ*, *IL1β*, and *CCL5*, was comparable between BMRKO-HFD and LC-HFD mice (Fig. 5E). Instead, we observed significantly increased expression of *IFNγ* and *CCL5* in eWAT of BMRKO-NCD mice compared with LC-NCD mice (Fig. 5E). Adipose tissue inflammation is often coupled with tissue fibrosis, which significantly affects the function of brown adipose (37). Masson’s trichrome staining displayed more fibrotic area in the eWAT of BMRKO-HFD mice compared with LC-HFD mice (supplemental Fig. S2A, B). In addition, profibrotic gene MMP2 significantly increased in BAT MRKO mice compared with LC mice under HFD (supplemental Fig. S2C).

### BAT MR deficiency aggravates hepatic steatosis in mice fed with HFD

**Fig. 6:**
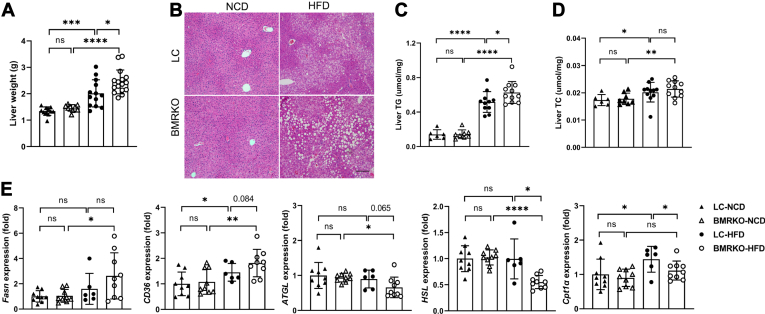
BAT MR deficiency aggravates hepatic steatosis in mice fed with HFD. A: Liver weight. B: Representative H&E staining of liver sections. Scale bar represents 100 μm. *n* = 11:9:13:16. C: Fasting liver triglycerides (TGs). *n* = 6:8:11:11. D: Fasting liver cholesterol (total cholesterol). *n* = 6:8:11:11. E: RT-qPCR analysis of *Fasn*, *CD36*, *ATGL*, *HSL*, and *Cpt1α* in liver samples. *n* = 9:9:6:9. Values are expressed as mean ± SD. ∗*P* < 0.05, ∗∗*P* < 0.01, ∗∗∗*P* < 0.001, and ∗∗∗∗*P* < 0.001. ns, not significant.

The liver weight of BMRKO-HFD mice was markedly higher than that of LC-HFD mice (Fig. 6A). The results of H&E staining illustrated that the liver of BMRKO-HFD mice developed more lipid droplets compared with LC-HFD mice (Fig. 6B). In addition, the hepatic triglyceride content was markedly increased in BMRKO-HFD mice compared with LC-HFD mice (Fig. 6C). There was no apparent difference in hepatic total cholesterol between LC-HFD and BMRKO-HFD mice (Fig. 6D). RT-qPCR results demonstrated that gene expression of *CD36*, which facilitates fatty acid intake, tended to be higher in livers of BMRKO-HFD mice than LC-HFD mice (Fig. 6E). Meanwhile, the expression of genes related to lipolysis, including *HSL* and *ATGL*, significantly decreased or showed a strong trend of decrease in livers of BMRKO-HFD mice (Fig. 6E). Gene expression of *Cpt1α*, which is critical for fatty acid oxidation, also significantly decreased in livers of BMRKO-HFD mice (Fig. 6E). The expression of all these genes was comparable between LC and BMRKO mice under NCD (Fig. 6E).

### BAT MR deficiency decreases the level of insulin-induced phosphorylation of AKT in eWAT of mice fed with HFD

**Fig. 7:**
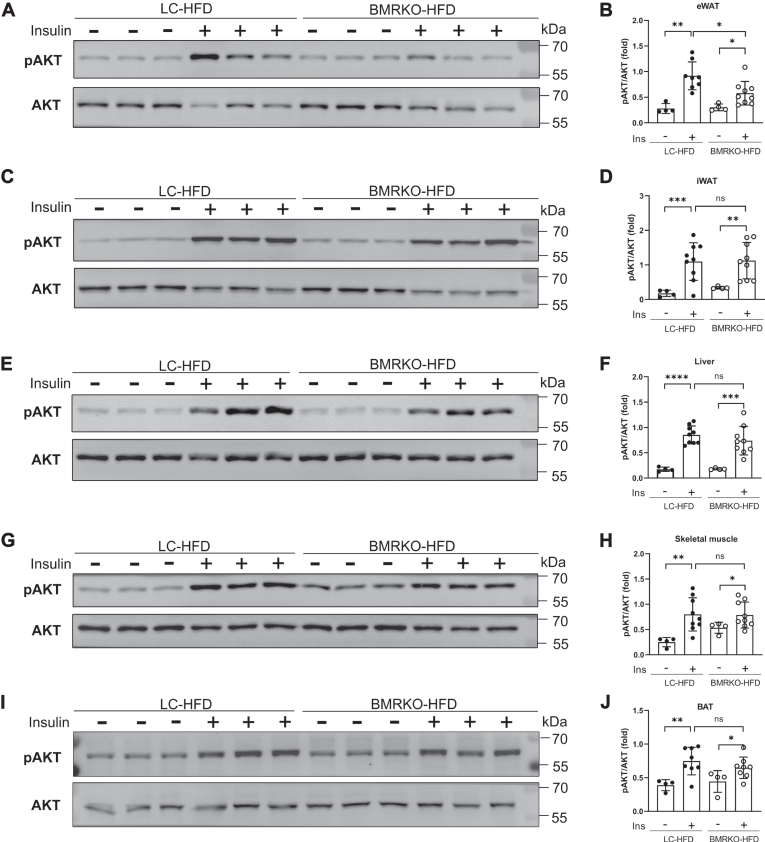
BATMR deficiency attenuates insulin-induced phosphorylation of Akt in eWAT of mice fed with HFD. A: Representative images of Western blotting of pAKT (Ser473) and total AKT in eWAT of mice fed with HFD for 8 weeks. B: Quantification of Western blotting results of eWAT. *n* = 4:8:4:9. C–J: Representative images of Western blotting analysis and quantifications of pAKT and total AKT in (C, D) iWAT, (E, F) liver, (G, H) skeletal muscle, and (I, J) BAT. *n* = 4:9:4:9 for iWAT, liver, and skeletal muscle. *n* = 4:8:4:8 for BAT. Values are expressed as mean ± SD. ∗*P* < 0.05, ∗∗*P* < 0.01, ∗∗∗*P* < 0.001, and ∗∗∗∗*P* < 0.001. ns, not significant.

**Supplemental Figure S3:**
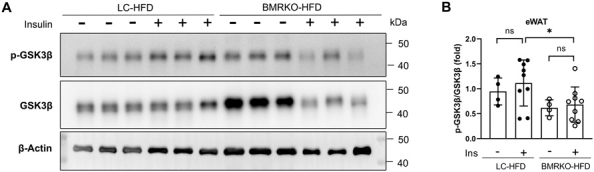
BAT MR deficiency attenuates insulin-induced phosphorylation of GSK-3β in eWAT of mice fed with HFD. A: Representative images of Western blotting of p-GSK3β (Ser9) and total GSK3β in eWAT of mice fed with HFD for 8 weeks. B: Quantification of Western blotting results of eWAT. *n* = 4:9:4:9. Values are expressed as mean ± SD. ∗*P* < 0.05. ns, not significant.

To determine the target organ responsible for insulin resistance, we tested the phosphorylation level of AKT (pAKT) in eWAT, iWAT, liver, skeletal muscle, and BAT 10 min after insulin injection in HFD-fed mice. Results of Western blotting illustrated that insulin-induced increase of pAKT was significantly attenuated in eWAT of BMRKO-HFD mice than that of LC-HFD mice (Fig. 7A, B). In other tissues, including iWAT, liver, skeletal muscle, and BAT, insulin-induced pAKT was similar between the two groups (Fig. 7C–J). In addition, significantly lower level of p-GSK3β was detected in eWAT of BMRKO-HFD mice than LC-HFD mice under insulin stimulation (supplemental Fig. S3A, B). These results indicated that impaired insulin sensitivity in eWAT may contribute to glucose intolerance of BMRKO-HFD mice.

## Discussion

Although the function of white adipocyte MR in metabolic regulation has been reported, the function of BAT MR in obesity and metabolic regulation has been unclear. The present study revealed that BAT MR deficiency exacerbated obesity-induced glucose intolerance and insulin resistance, particularly in eWAT. Mice with BAT MR deficiency also demonstrated a decreasing trend of energy metabolism, aggravated eWAT inflammation, and hepatic steatosis.

We have demonstrated that BAT MR deficiency worsens obesity and related metabolic dysregulation. MR antagonists, including spironolactone (the first generation), eplerenone (the second generation), and finerenone (the third and newest generation), have all been reported to alleviate obesity-induced metabolic disorders (20, 38, 39). Interestingly, recent data have shown that finerenone, but not spironolactone, is able to improve the function of BAT specifically (not WAT) and increase the recruitment of brown adipocytes (39, 40). However, the function of adipocyte MR in metabolic regulation is not that conclusive according to the results from the studies using adipocyte MRKO mice (23, 24, 25). Although deletion of MR in both WAT and BAT alleviates obesity-induced hepatic steatosis in mice, it has no or only minor impacts on glucose intolerance and insulin resistance (23, 24, 25). In contrast, our results showed that deletion of MR in BAT (but not WAT) significantly exacerbated obesity, glucose intolerance, hepatic steatosis, and eWAT inflammation and fibrosis in obese mice. The difference of metabolic disorder between BAT-specific MR KO and MR antagonist may suggest different regulatory mechanisms. Future work that delicately dissects the role of MR in WAT and BAT specifically may provide more insights to comprehend the function of MR in metabolic regulation.

BAT MR deficiency affects gene alteration related to lipid metabolism in BAT. Our data showed more lipid accumulation in BAT but no change in energy metabolism in BMRKO mice. Furthermore, BMRKO led to decreased expression of *Acot1*, *Acot4*, *Acsm3*, *Hsd11β1*, *Ehhadh*, *Ucp3*, *Slc27a2*, *Dagla*, and *Slc22a5* and increased expression of *Serpine1* in BAT, probably pointing to reduce fatty acid or lipid metabolism. BAT-driven nonshivering thermogenesis is an essential mechanism that promotes EE and ultimately metabolic health (41). A lot of efforts have been made to enhance the function of BAT to increase EE and fight against obesity-related metabolic disorders (42). It is likely that MR deficiency affects metabolic homeostasis probably by lipid metabolism-related machinery in BAT. In this context, BAT MR is a potential target for manipulations of lipid metabolism in BAT.

WAT may have mediated the deleterious effects of BAT MR deficiency under obesity. Overactivation of MR suppresses the phosphorylation of PI3K/AKT and leads to insulin resistance (43), whereas blockade of MR enhances insulin sensitivity in obese mice (21), suggesting that MR is critical in the pathogenesis of insulin resistance. Several lines of reports have elucidated that immune cells are involved in the development of insulin resistance (44, 45). Obesity promotes macrophage proliferation and triggers inflammation in adipose tissue (46). Inhibition of proinflammatory cytokines *TNFa*, *IL1β*, or *MCP1* reverses metabolic disorders (47). Transplantation of BAT function has been demonstrated to attenuate inflammation of eWAT, leading to improved metabolic phenotype in an obese mouse model (48). Similarly, our results showed that manipulation of BAT function by BMRKO aggravated inflammation and insulin resistance in eWAT of obese mice. Therefore, it is plausible that BAT MR deficiency leads to metabolic disturbance in WAT and eventually systemic dysregulation of metabolism. We exploited a constitutive KO strategy to delete MR in BAT of our mouse model. It remains to be further determined whether such strategy affects the development of mice during the fetal and prenatal stages and whether this affects the interpretation of the metabolic phenotypes.

In conclusion, BAT MR plays a crucial role in the regulation of obesity and related metabolic disorders. The findings have unveiled novel functions of BAT MR and support that perturbation of MR in BAT specifically may provide therapeutic strategies for obesity-related metabolic diseases.

## Data availability

All relevant data generated during the present study and its supplemental data are contained within the main text. The raw data of RNA sequencing have been deposited in online repositories. The names of the repository/repositories and accession number(s) can be found below: https://www.ncbi.nlm.nih.gov/bioproject/?term=PRJNA795574.

## Supplemental data

This article contains supplemental data.

## Conflict of interest

The authors declare that they have no conflicts of interest with the contents of this article.
