# Supplementary material for: Brown adipocyte mineralocorticoid receptor deficiency impairs metabolic regulation in diet-induced obese mice
Source: J Lipid Res. 2023 Sep 20;64(11):100449. doi: 10.1016/j.jlr.2023.100449 (PMC10622702; doi:10.1016/j.jlr.2023.100449)
Supplement: Supplemental Table S1 [file mmc1.docx]

**Table S1. Primers presented in the study.**

| **Genes** | **Forward 5’-3’** | **Reverse 5’-3’** |
| --- | --- | --- |
| ACSM3 | CTTTGGCCCCAGCAGTAGATG | GGCTGTCACTGGCATATTTCAT |
| ACOT1 | ATACCCCCTGTGACTATCCTGA | CAAACACTCACTACCCAACTGT |
| ATGL | GGATGGCGGCATTTCAGACA | CAAAGGGTTGGGTTGGTTCAG |
| CCL5 | GCTGCTTTGCCTACCTCTCC | TCGAGTGACAAACACGACTGC |
| CD36 | ATGGGCTGTGATCGGAACTG | GTCTTCCCAATAAGCATGTCTCC |
| Cpt1α | CTCCGCCTGAGCCATGAAG | CACCAGTGATGATGCCATTCT |
| Fasn | GGAGGTGGTGATAGCCGGTAT | TGGGTAATCCATAGAGCCCAG |
| GAPDH | AGGTCGGTGTGAACGGATTTG | TGTAGACCATGTAGTTGAGGTCA |
| HSL | CCAGCCTGAGGGCTTACTG | CTCCATTGACTGTGACATCTCG |
| INFγ | TCTGAGACAATGAACGCTAC | CTTCCACATCTATGCCACTT |
| IL-1β | AAGAGCTTCAGGCAGGCAGTATCA | TGCAGTTGTCTAATGGGAACGTCA |
| MCP1 | TGAAGTTGACCCGTAAATCTGA | CATGAAAGGGAATACCATAACA |
| MR | TCCAAGATCTGCTTGGTGTGTGGA | AGGCAGGACAGTTCTTTCTCCGAA |
| Serpine1 | TTCAGCCCTTGCTTGCCTC | ACACTTTTACTCCGAAGTCGGT |
| Slc27a2 | TCCTCCAAGATGTGCGGTACT | TAGGTGAGCGTCTCGTCTCG |
| TNFα | CCCCAGTCTGTATCCTTCTAAC | TCACTGTCCCAGCATCTTGT |
| UCP3 | CTGCACCGCCAGATGAGTTT | ATCATGGCTTGAAATCGGACC |
| UCP1 | AGGCTTCCAGTACCATTAGGT | CTGAGTGAGGCAAAGCTGATTT |
| Nduf8 | TGTTGCCGGGGTCATATCCTA | AGCATCGGGTAGTCGCCATA |
| SDHB | AATTTGCCATTTACCGATGGGA | AGCATCCAACACCATAGGTCC |
| Uqcrc1 | AGACCCAGGTCAGCATCTTG | GCCGATTCTTTGTTCCCTTGA |
| Uqcrc2 | AAAGTTGCCCCGAAGGTTAAA | GAGCATAGTTTTCCAGAGAAGCA |
| Cox4 | ATTGGCAAGAGAGCCATTTCTAC | CACGCCGATCAGCGTAAGT |
| ATP5a1 | TCTCCATGCCTCTAACACTCG | CCAGGTCAACAGACGTGTCAG |
| Col1a1 | GCTCCTCTTAGGGGCCACT | CCACGTCTCACCATTGGGG |
| Col6a1 | CTGCTGCTACAAGCCTGCT | CCCCATAAGGTTTCAGCCTCA |
| Mmp2 | CAAGTTCCCCGGCGATGTC | TTCTGGTCAAGGTCACCTGTC |
